# Abnormal RasGRP1 Expression in the Post-Mortem Brain and Blood Serum of Schizophrenia Patients

**DOI:** 10.3390/biom12020328

**Published:** 2022-02-18

**Authors:** Arianna De Rosa, Anna Di Maio, Silvia Torretta, Martina Garofalo, Valentina Giorgelli, Rita Masellis, Tommaso Nuzzo, Francesco Errico, Alessandro Bertolino, Srinivasa Subramaniam, Antonio Rampino, Alessandro Usiello

**Affiliations:** 1 Laboratory of Translational Neuroscience, CEINGE Biotecnologie Avanzate, 80145 Naples, Italy; derosaar@ceinge.unina.it (A.D.R.); dimaio@ceinge.unina.it (A.D.M.); garofalom@ceinge.unina.it (M.G.); nuzzo@ceinge.unina.it (T.N.); erricof@ceinge.unina.it (F.E.); 2 Group of Psychiatric Neuroscience, Department of Basic Medical Sciences, Neuroscience and Sense Organs, University of Bari Aldo Moro, 70124 Bari, Italy; torretta.silvia@gmail.com (S.T.); valegio3@gmail.com (V.G.); rita.masellis@uniba.it (R.M.); alessandro.bertolino@uniba.it (A.B.); 3 Department of Environmental, Biological and Pharmaceutical Science and Technologies, Università degli Studi della Campania “Luigi Vanvitelli”, 81100 Caserta, Italy; 4 Department of Agricultural Sciences, University of Naples “Federico II”, 80055 Naples, Italy; 5 Azienda Ospedaliero Universitaria Consorziale Policlinico, 70124 Bari, Italy; 6 Department of Neuroscience, The Scripps Research Institute, Jupiter, FL 33458, USA; ssubrama@scripps.edu

**Keywords:** schizophrenia, RasGRP1, DLPFC, hippocampus, serum

## Abstract

Schizophrenia (SCZ) is a polygenic severe mental illness. Genome-wide association studies (GWAS) have detected genomic variants associated with this psychiatric disorder and pathway analyses have indicated immune system and dopamine signaling as core components of risk in dorsolateral-prefrontal cortex (DLPFC) and hippocampus, but the mechanistic links remain unknown. The *RasGRP1* gene, encoding for a guanine nucleotide exchange factor, is implicated in dopamine signaling and immune response. *RasGRP1* has been identified as a candidate risk gene for SCZ and autoimmune disease, therefore representing a possible point of convergence between mechanisms involving the nervous and the immune system. Here, we investigated RasGRP1 mRNA and protein expression in post-mortem DLPFC and hippocampus of SCZ patients and healthy controls, along with RasGRP1 protein content in the serum of an independent cohort of SCZ patients and control subjects. Differences in RasGRP1 expression between SCZ patients and controls were detected both in DLPFC and peripheral blood of samples analyzed. Our results indicate RasGRP1 may mediate risk for SCZ by involving DLPFC and peripheral blood, thus encouraging further studies to explore its possible role as a biomarker of the disease and/or a target for new medication.

## 1. Introduction

Schizophrenia (SCZ) is a severe brain disorder affecting about 1% of the population worldwide. It is characterized by a complex clinical phenotype, which includes positive symptoms, such as delusions and hallucinations, negative symptoms, such as blunted affect, anhedonia, social withdrawal and apathy, along with cognitive impairment [1]. Evidence robustly indicates that the risk for SCZ is, at least in part, genetic and that genetic variation associated with the disorder likely subtends different pathophysiological mechanisms. During last few decades, a number of pathogenetic hypotheses of SCZ have been formulated, including the “dopamine hypothesis” [2]. According to this hypothesis, altered transmission mediated by D2 and D1 dopamine receptors in critical brain regions, such as the hippocampus and the Prefrontal Cortex (PFC), have been reported in patients with this mental illness [3]. Nonetheless, several hypotheses, in part implicating other brain signaling systems, such as the glutamate, GABA and serotonin systems [4,5,6,7,8], in part implicating neurodevelopment and inflammation, have been also proposed. In the recent past, pathogenetic pathways of SCZ implicating alteration of the immune system, along with inflammatory processes operating in the brain (neuro-inflammation) and in the body periphery, have been claimed. Consistently, genome wide association studies (GWASs) have indicated that genomic variation with the strongest association with SCZ is localized within the major histocompatibility complex II (MHC-II) locus, a large segment of the genome with a complex genetic architecture, which has only partially been clarified [9]. Furthermore, patients with SCZ suffer from infectious and autoimmune diseases more often than the general population and genetic risk factors for SCZ increase vulnerability to these diseases [10]. Secondly, prenatal complication, which increases risk for SCZ [11], also impairs adult life immune system competence. Moreover, while immune dysfunctions, such as imbalances of pro- and anti-inflammatory cytokines, may contribute to the onset of psychotic symptoms, many antipsychotic (AP) medications have proven anti-infectious and immune-modulating effects in SCZ patients [10,12]. Interestingly, evidence from GWASs of immune system diseases, such as autoimmune thyroid disease, celiac disease, Crohn’s disease, inflammatory bowel disease, psoriasis, rheumatoid arthritis (RA), Sjögren’s syndrome and systemic lupus erythematosus (SLE), indicates that, even at different levels of significance, these conditions are genetically correlated with SCZ [12,13]. Again, this evidence suggests that molecular pathways subtending SCZ, and immune disorders are, at least in part, overlapping and that risk genes for SCZ may have a pleiotropic effect on risk for immune dysfunction [14]. With this regard, converging findings have indicated that the gene coding for the guanine-nucleotide-exchange factor 1 (RASGRP1), an enzyme that catalyzes the exchange of a G-protein bound guanosine diphosphate (GDP) to guanosine triphosphate (GTP) in manifold biochemical processes [15] and whose locus is genome-wide associated with SCZ and RA [16], is a putative link between SCZ and alteration of the immune system. RasGRP1 that belongs to a group of proteins functioning as guanine nucleotide exchange factors (GEFs) highly expressed in hematopoietic cells is known to play a role in T and B cell proliferation and has been implicated in leukemia and lupus erythematosus [17,18,19].

Furthermore, this protein is also involved in immunodeficiency, immune dysregulation and in Epstein–Barr Virus-induced Lymphoma [20]. Nonetheless, besides blood cells, RasGRP1 is enriched in specific brain regions where it regulates brain signaling of established importance to SCZ, such as dopamine D1-mediated cognitive function [21] along with extracellular signal-regulated kinases (ERK) and mechanistic target of rapamycin kinase (mTOR) molecular signaling [22]. In addition, recent evidence reports that *RasGRP1* belongs to a preserved co-expression module of SCZ-risk genes functionally characterized by their contributing to rat sarcoma virus (RAS)/ERK signaling [23]. Finally, an investigation performed to identify genome wide genetic variation associated with SCZ enriched for association with RA, one of the most severe immune system diseases, has indicated *RasGRP1* as a strong link between genetic risk for SCZ and this autoimmune condition [16]. Therefore, by playing a role in both dopamine neurotransmission and immune processes of relevance to SCZ, RasGRP1 may be a point of convergence of risk at the crossroad between the central nervous system and the peripheral blood/immune system, thus representing a possible peripheral biomarker of the illness. In order to probe this hypothesis, we measured the RasGRP1 mRNA and protein expression in post-mortem dorsolateral prefrontal cortex (DLPFC) and the hippocampus of patients with SCZ compared to healthy controls. Moreover, we analyzed RasGRP1 protein serum concentration in an independent cohort of SCZ patients and non-psychiatric controls. Differences in RasGRP1 expression between SCZ patients and controls were detectable both in the brain and in peripheral blood of samples explored.

## 2. Materials and Methods

### 2.1. Human Post-Mortem Tissue Collection

Human DLPFC and hippocampus samples were collected from post-mortem brains of non-psychiatric controls and SCZ patients (*n* = 20/brain region/clinical condition). Samples were obtained from The Human Brain and Spinal Fluid Resource Center (Los Angeles Healthcare Center, Los Angeles, CA, USA). Clinical diagnosis of SCZ was performed according to DSM III-R criteria. Demographic characteristics of control and SCZ subjects are described in Table 1 and Table S1, Supplementary Materials. Frozen tissues were pulverized in liquid nitrogen and stored at −80 °C for subsequent processing.

### 2.2. RNA Extraction and Quantitative RT-PCR Analysis

Total RNA was extracted from post-mortem tissues using RNeasy^®^ mini kit (Qiagen, Hilden, Germany) according to the manufacturer’s instructions [24,25]. Total RNA was purified to eliminate potentially contaminating genomic DNA using recombinant DNase (Qiagen, Hilden, Germany). RNA integrity number (RIN) of samples was assessed using Agilent Expert version 2100 (Santa Clara, CA, USA) [26] and Biorad Automated electrophoresis Station Experion Software version 3.20 (Hercules, CA, USA) [27] prior to cDNA synthesis using a Transcriptor First Strand cDNA Synthesis kit (Roche Diagnostics, Mannheim, Germany). A total of 1 μg of total RNA of each sample was reverse transcribed with QuantiTect Reverse Transcription (Qiagen, Hilden, Germany) using oligo-dT and random primers according to the manufacturer’s instructions. Quantitative RT-PCR with Real Time ready catalogue Assays (Roche Diagnostics, Basel, Switzerland) and LightCycler^®^ 480 Probe Master (Roche Diagnostics) was performed on a Light Cycler 480 Real Time PCR thermocycler with 96-well format (Roche Diagnostics). All measurements from each subject were performed in duplicate. *RasGRP1* mRNA expression levels were normalized to the mean of two housekeeping genes: β-actin (*ACTB*) and cyclophilin (*PPIA*). The following protocol was used: 10 s for initial denaturation at 95 °C followed by 40 cycles consisting of 10 s at 94 °C for denaturation, 10 s at 60 °C for annealing, and 6 s for elongation at 72 °C temperature. The following primers were used for *RasGRP1* cDNA amplification: *RasGRP1* forward, *5′*-GGA GGC TAA CAA GGA CTT GGT AC-*3′* and *RasGRP1* reverse, *5′*-GGT GGC TTTGAA GGT GTT AGT GG-*3′*; *β-actin* forward, *5′*-TCCTCCCTGGAGAAGAGCTA-*3′*; *β-actin* reverse, *5′*-CGTGGATGCCACAGGACT-*3′*; *PPIA* forward, *5′*-TTCATCTGCACTGCCAAGAC-*3′*; *PP1A* reverse, *5′*-CACTTTGCCAAACACCACAT-*3′*. qPCR assay detects all the transcripts of *RasGRP1* gene. mRNA expression was calculated using the geometric mean of the two reference genes selected and the relative quantification method (2^−ΔΔCt^).

### 2.3. Western Blotting

Frozen, powdered samples from post-mortem DLPFC and hippocampus and from SFG of respective brain banks were sonicated in 1% SDS and boiled for 10 min. Aliquots (2 µL) of the homogenate were used for protein determination using a Bio-Rad Protein Assay kit. Equal amounts of total proteins (30 µg) for each sample were loaded on precast 4–20% gradient gels (BioRad Laboratories, Hercules, CA, USA). Proteins were separated by SDS-PAGE and transferred to PVDF membranes (GE Healthcare, Chicago, IL, USA) via the Trans Blot Turbo System (BioRad Laboratories, Hercules, CA, USA). To investigate the target of interest, the blots were incubated with antibodies against: RasGRP1 (1:1000; no. MABS146, Merck Millipore, Burlington, MA, USA). GAPDH (1:1000; sc-32233, Santa Cruz Biotechnology, Dallas, TX, USA) was used to normalize the levels of analyzed proteins for variations in loading and transfer. All blots were incubated in horseradish peroxidase-conjugated secondary antibodies and target proteins visualized by ECL detection (Pierce, Rockford, IL, USA), followed by quantification through the “Quantity One” software (BioRad Laboratories, Hercules, CA, USA). Normalized values were then averaged and used for statistical comparisons.

### 2.4. Human Patient and Control Serum Collection

A group of 40 outpatients with SCZ and 39 healthy control individuals were recruited in the region of Apulia, Italy. Recruitment procedures were carried out in accordance with The Code of Ethics of the World Medical Association (Declaration of Helsinki), and approval was given by the local ethics committee (“Comitato Etico Indipendente Locale—Azienda Ospedaliero-Universitaria Consorziale Policlinico di Bari”). In patients (males = 22; females = 18; Median Age = 33.5), the diagnosis of SCZ was made using the Structured Clinical Interview for the DSM-5 (SCID), Axis 1 disorders (Diagnostic and Statistical Manual of Mental Disorders: DSM-5. Arlington, VA: American Psychiatric Publishing, 2013), which was administered by psychiatrists. Healthy individuals (males = 15; females = 24; Median Age = 28) underwent the NP (non-patient) version of the SCID in order to exclude the presence of any psychiatric disorders. Both patients and healthy controls were excluded if they had: a significant history of drug or alcohol abuse; active drug abuse in the previous year; experienced a head trauma with a loss of consciousness; or if they suffered from any other significant medical condition. All participants in the study underwent 10 mL whole blood withdrawal for subsequent serum isolation. Socio-demographics of serum study sample are reported in Table 2.

### 2.5. Enzyme Linked Immunosorbent Assay (ELISA)

RasGRP1 concentration was detected using Human RAS Guanyl-Releasing Protein 1 (RASGRP1) ELISA Kit according to the manufacturer’s instructions (#MBS9331756, MyBiosource, San Diego, CA, USA). Serum was collected and centrifuged at 3000 rpm for 20 min, and the supernatant was collected. In addition, a 50 μL serum was added to every sample well and 50 μL Standard to corresponding Standard wells. Next, 100 μL HRP-Conjugate Reagent was added to every well. After 60 min of incubation at 37 °C, all wells were washed 4 times. Furthermore, 50 μL Chromogen Solution A and 50 μL Chromogen Solution B were added to every well. After 15 min of incubation at 37 °C, 50 μL of Stop Solution was added to every well. The Optical Density (O.D.) was readied at 450 nm using an ELISA reader within 15 min after adding Stop Solution. The sensitivity of this kit is 1.0 ng/mL.

### 2.6. Statistical Analysis

Data are reported as medians, along with interquartile range (first-third quartiles—IQR). Differences in continuous variables among two groups were evaluated by an Unpaired *t*-test. Asterisks denote statistical significance as calculated by the specific statistical tests (* *p* < 0.05). Pearson’s correlation was used to test possible associations between two variables. To explore associations between RasGPR1 mRNA and protein expression and different variables, after adjustment for possible confounding factors (age and/or PMI), logistic regression models were used. Box and whiskers plot were used to depict statistically significant differences between groups.

## 3. Results

### 3.1. RasGRP1 Protein Levels Negatively Correlate with Age in the DLPFC of Schizophrenia Patients

Firstly, we evaluated whether the two clinical groups, healthy subjects and schizophrenia patients, were matched for demographic and post-mortem storage characteristics, including gender, age, PMI and samples’ pH. Data indicated that subjects were matched for gender (*p* = 0.301, *Chi-Square* test) and pH (*p* = 0.485; two-sample *t*-test on log values). Conversely, we observed a significant difference between patient and control age (*p* < 0.001, two-sample *t*-test) and PMI (*p* = 0.020, two-sample *t*-test on log values). Consistent with this observation, we investigated the correlations between RasGRP1 mRNA and protein expression levels with age and PMI in the DLPFC of SCZ patients and healthy controls.

**Figure 1:**
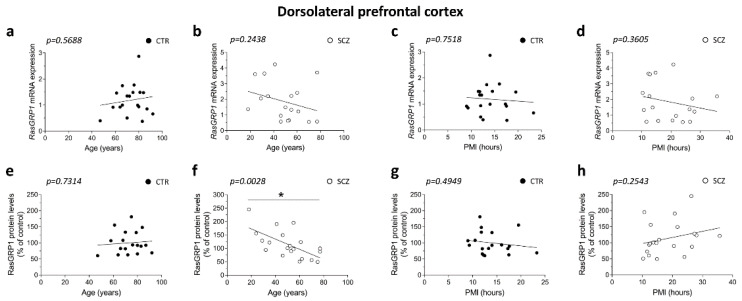
RasGRP1 protein levels negatively correlates with age but not with PMI in the DLPFC of schizophrenia patients. Analysis of correlation between age and mRNA/protein levels of RasGRP1 in the post-mortem DLPFC of (**a**,**e**) control subjects (CTR, *n* = 19) and (**b,f**) SCZ patients (SCZ, *RasGRP1* mRNA: *n* = 18, RasGRP1 protein: *n* = 20). Analysis of correlation between PMI with mRNA and protein levels of RasGRP1 in the DLPFC of (**c**,**g**) control subjects (CTR, *n* = 19) and (**d**,**h**) SCZ patients (SCZ, *RasGRP1* mRNA: *n* = 18, RasGRP1 protein: *n* = 20).

Results showed that there was no correlation between RasGRP1 expression and age in the control group (*RasGRP1* mRNA: Pearson’s *r* = 0.1395; *p* = 0.5688; RasGRP1 protein: Pearson’s *r* = 0.08432; *p* = 0.7314), (Figure 1a,e). Moreover, we failed to observe any significant correlation between *RasGRP1* mRNA and age in SCZ patients (Pearson’s *r* = −0.2895; *p* = 0.2438) (Figure 1b), while discovering that RasGRP1 protein levels negatively correlated with age in the DLPFC of SCZ patients (Pearson’s *r* = −0.6311; *p* = 0.0028) (Figure 1f). Next, we investigated the correlation between PMI and RasGRP1 expression in the same brain region. No correlations were observed between RasGRP1 mRNA or protein levels and PMI in the DLPFC of control subjects (*RasGRP1* mRNA: Pearson’s *r* = −0.07773; *p* = 0.7518; RasGRP1 protein: Pearson’s *r* = −0.1668; *p* = 0.4949), (Figure 1c,g). Likewise, in SCZ patients, we did not find any significant correlation between RasGRP1 expression and PMI (*RasGRP1* mRNA: Pearson’s *r* = −0.2291; *p* = 0.3605; RasGRP1 protein: Pearson’s *r* = 0.2675; *p* = 0.2543) (Figure 1d,h).

### 3.2. Increased RasGRP1 mRNA Levels in the DLPFC of Patients with SCZ

**Figure 2:**
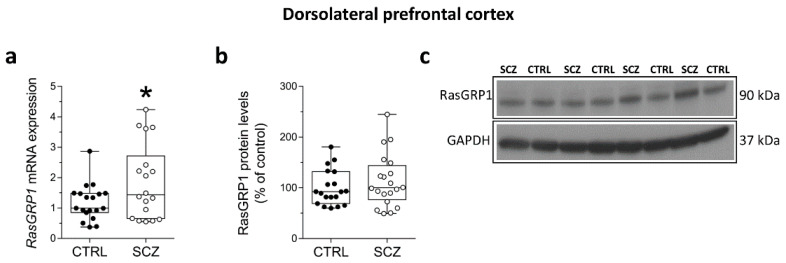
Increased *RasGRP1* mRNA levels in the DLPFC of patients with SCZ. Expression levels of (**a**) *RasGRP1* mRNA in the post-mortem DLPFC of SCZ patients (SCZ) (*n* = 18) and control subjects (CTRL) (*n* = 19). Quantification of western blot analysis of (**b**) RasGRP1 protein levels in the total homogenates of post-mortem DLPFC of SCZ-affected patients (SCZ) (*n* = 20) and control subjects (CTRL) (*n* = 19). The variations of RasGRP1 protein levels in patients affected by SCZ are expressed as percentage (%) of the control subjects. All markers were normalized to GAPDH for variation in loading and transfer; (**c**) representative images of immunoblots of RasGRP1 performed in the DLPFC of SCZ patients and control subjects. Each dot represents value from a single subject. * *p* < 0.05 compared to control group (Unpaired *t*-test). Multivariable regression analysis was performed against age and post-mortem interval (PMI).

Although with a relatively small effect, statistical analysis indicated an increase of *RasGRP1* transcript levels in the DLPFC of SCZ patients compared to non-psychiatric controls (*p* = 0.0395; Unpaired *t*-test; adjusted *p* = 0.046; multivariate logistic regression) (Figure 2a). Results also indicated comparable protein levels between patients and controls (*p* = 0.3386; Unpaired *t*-test), (Figure 2b).

### 3.3. Lack of Correlation between RasGRP1 mRNA and Protein Levels with Age and PMI in the Hippocampus of Schizophrenia Patients

**Figure 3:**
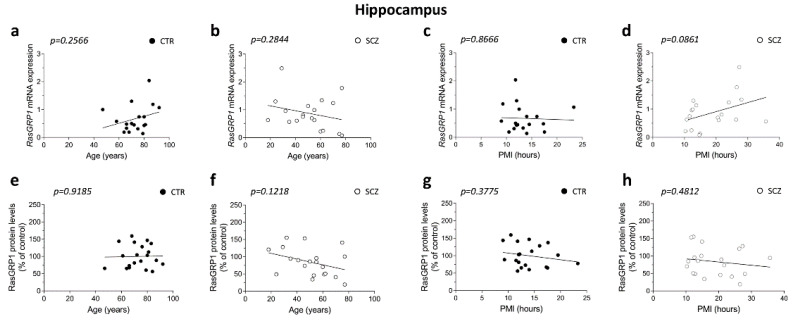
Lack of correlation between RasGRP1 mRNA and protein levels with age and PMI in the hippocampus of schizophrenia patients. Analysis of correlation between age and mRNA and protein levels of RasGRP1 in post-mortem hippocampus of (**a**,**e**) control subjects (CTR, *RasGRP1* mRNA: *n* = 18, RasGRP1 protein: *n* = 20) and (**b**,**f**) SCZ patients (SCZ, *RasGRP1* mRNA: *n* = 19, RasGRP1 protein: *n* = 20). Analysis of correlation between PMI with mRNA and protein levels of RasGRP1 in DLPFC of (**c**,**g**) control subjects (CTR, *RasGRP1* mRNA: *n* = 18, RasGRP1 protein: *n* = 20) and (**d**,**h**) SCZ patients (SCZ, *RasGRP1* mRNA: *n* = 19, RasGRP1 protein: *n* = 20).

We extended our study by exploring possible correlation between age and PMI with *RasGRP1* mRNA and protein levels in the hippocampus. Overall, we failed to find any significant correlation between RasGRP1 expression and age in both non-psychiatric individuals and SCZ patients (CTR: RasGRP1 mRNA: Pearson’s *r* = 0.2822; *p* = 0.2566; RasGRP1 protein: Pearson’s *r* = 0.02446; *p* = 0.9185; *n* = 20; SCZ: *RasGRP1* mRNA: Pearson’s *r* = −0.2589; *p* = 0.2844; RasGRP1 protein: Pearson’s *r* = −0.3574; *p* = 0.1218), (Figure 3a,b,e,f). Likewise, no correlations were observed between RasGRP1 mRNA or protein levels with PMI in the hippocampus of both groups (CTR: *RasGRP1* mRNA: Pearson’s *r* = −0.04262; *p* = 0.8666; RasGRP1 protein: Pearson’s *r* = −0.2086; *p* = 0.3775; SCZ: *RasGRP1* mRNA: Pearson’s *r* = 0.4042; *p* = 0.0861; RasGRP1 protein: Pearson’s *r* = −0.1671; *p* = 0.4812), (Figure 3c,d,g,h).

### 3.4. Unaltered RasGRP1 mRNA and Protein Levels in the Hippocampus of Schizophrenia Patients

**Figure 4:**
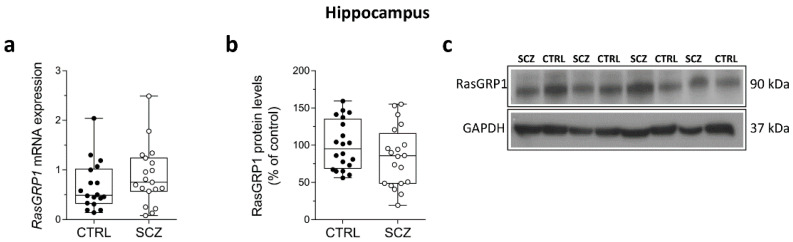
Unaltered RasGRP1 mRNA and protein levels in the hippocampus of schizophrenia patients. Expression levels of (**a**) *RasGRP1* mRNA in the post-mortem hippocampus of SCZ-affected patients (SCZ) (*n* = 19) and control subjects (CTRL) (*n* = 18). Quantification of western blot analysis of (**b**) RasGRP1 protein levels in the total homogenates of post-mortem hippocampus of SCZ-affected patients (SCZ) (*n* = 20) and control subjects (CTRL) (*n* = 20). The variations of RasGRP1 protein levels in patients affected by SCZ are expressed as percentage (%) of the control subjects. All markers were normalized to GAPDH for variations in loading and transfer; (**c**) representative images of immunoblots of RasGRP1 performed in the hippocampus of SCZ patients and control subjects. Each dot represents value from a single subject. Unpaired *t*-test was performed in all analyses reported.

We evaluated RasGRP1 mRNA and protein levels in the hippocampus of SCZ patients compared to control subjects. We did not find any significant difference in *RasGRP1* transcript levels in SCZ patients compared to non-psychiatric individuals (*p* = 0.2720; Unpaired *t*-test), (Figure 4a). Similarly, biochemical experiments failed to find a significant difference of RasGRP1 protein levels between SCZ patients and control subjects (*p* = 0.1821; Unpaired *t*-test) (Figure 4b).

### 3.5. Detection of RasGRP1 Protein Concentration in the Serum of Healthy Controls and Schizophrenia Patients

**Figure 5:**
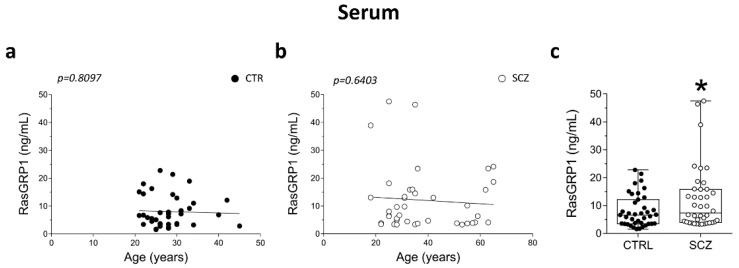
Detection of RasGRP1 protein concentration in the serum of healthy controls and schizophrenia patients. Analysis of correlation between age and RasGRP1 serum concentration of (**a**) control subjects (CTR, *n* = 39) and (**b**) SCZ patients (SCZ, *n* = 40). Analysis of (**c**) RasGRP1 concentration (ng/mL) in serum of SCZ-affected patients (SCZ, *n* = 40) and control subjects (CTR, 39). * *p* < 0.05 compared to control group (Unpaired *t*-test). Multivariable regression analysis was performed against age.

Here, we determined serum levels of RasGRP1 by ELISA analysis in a group of SCZ patients (*n* = 40) and control individuals (*n* = 39) (clinical and demographic characteristics shown in Table 2). Possible confounding effect of age on RasGRP1 protein concentration in serum was assessed by performing correlation analysis between age and RasGRP1 concentration of an independent cohort of SCZ patients and control subjects. We did not find any significant correlation between RasGRP1 content and age in the control group (Pearson’s *r* = −0.03983; *p* = 0.8097), (Figure 5a) and SCZ patients (Pearson’s *r* = −0.0762; *p* = 0.6403) (Figure 5b). However, analysis showed a strong statistical trend (*p* = 0.0497, Unpaired *t*-test; adjusted *p* = 0.050; multivariate logistic regression) indicating an increase of RasGRP1 concentration in serum of SCZ patients compared to healthy controls (Figure 5c).

## 4. Discussion

Different lines of research, including single gene and GWAS, co-expression, and pathway analyses, along with pre-clinical studies converge on indicating *RasGRP1* as a risk gene for SCZ [16,23]. Consistent with this evidence, RasGRP1 modulates dopamine signaling downstream of D1R activation via cAMP/PKA, mTOR/ERK pathways in the medium spiny neurons of caudate putamen [22,24], as well as regulates synaptic plasticity in the hippocampus [28]. Moreover, this protein also affects immune system processes [19,29] potentially implicated in pathophysiology of SCZ [30]. In keeping with this, the *RasGRP1* gene has been recently indicated by genome wide investigation as a point of risk convergence between SCZ and immune system diseases [16]. Based on this evidence, here we investigated mRNA and protein levels of RasGRP1 in post-mortem DLPFC and hippocampus of patients with SCZ and healthy controls. Furthermore, we measured RasGRP1 protein concentration in serum of an independent sample of patients with SCZ and healthy individuals. Our results indicate abnormal greater transcript levels of *RasGRP1* in the DLPFC of SCZ patients compared to controls. Conversely, comparable protein levels in both hippocampus and DLPFC of SCZ patients and controls. The enhanced RasGRP1 mRNA but not protein levels in the DLPFC patients may indicate a tight translational control of RasGRP1, but mechanisms are still unknown. Moreover, we document a negative correlation between RasGRP1 and age selectively in DLPFC of SCZ patients. Furthermore, besides the brain, RasGRP1 is highly expressed in the blood cells, such as T-cells [31]. Interestingly, we found that RasGRP1 protein concentration was significantly higher in serum of patients with SCZ than healthy controls. Overall, we suggested RasGRP1 alteration in brain and in peripheral districts of the body in patients with this mental illness. To our knowledge, this is the first study that, although not providing any mechanistic insight into the role of RasGRP1 in pathophysiology of SCZ, highlights a possible link between this protein and the neurobiology subtending the disease. Recent findings have suggested a protein-protein interaction of RasGRP1 with the GTPase [32], Ras homolog enriched in striatum (Rhes), which had previously been implicated in SCZ [33,34] and whose prefrontal cortex expression is associated with phenotypes of relevance to the disorder [35]. Moreover, to further support the involvement of RasGRP1 in psychiatric illness, pilot studies in lymphoblastoid cells have indicated peripheral mRNA expression of *RasGRP1* to be a promising biomarker of different psychotic disorders, such as bipolar disorder [36], whose genetic background is largely shared with SCZ [37]. Additionally, compelling investigations have indicated RasGRP1 as a key molecule involved in peripheral immune processes of relevance to pathophysiology of severe immune diseases, including RA [38]. Based on the evidence that RA has a different incidence in patients with SCZ compared to the general population and shares a genetic risk with SCZ, we cannot rule out a potential role of RasGRP1 as a vulnerability factor for SCZ via its involvement in immune system. Intriguingly, some lines of research converge on this view. The involvement of *RasGRP1* expression in neuroinflammation is reported to be regulated by the Transcription Factor NR4A2 (Nurr1), which is also implicated in regulation of SCZ-risk gene co-expression in PFC [39]. Moreover, *RasGRP1* is among genes co-expressed in the PFC with genes contributing to risk for SCZ via ERK/mTOR signaling [40]. These pathways also promote inflammatory and immune functions, which are altered in SCZ [30,41]. Therefore, an increase in serum RasGRP1 may mirror inflammatory conditions and immune response in SCZ patients.

Notably, our findings indicate that altered *RasGRP1* transcript levels in SCZ are limited to the DLPFC and not extended to the hippocampus. The exact reason of such an observation is not clear; it may indicate a potential cell/tissue-specific regulation of *RasGRP1* gene expression in the brain. Previous reports have indicated RasGRP1 to be expressed in different brain areas [22,42,43,44], including the hippocampus, where its mRNA expression is implicated in synaptic plasticity, memory formation [43] and the cortex, where it is upregulated in mice chronically exposed to stress [45], a condition that has previously been associated with SCZ [46]. Nonetheless, further investigation into the differential role of RasGRP1 in different areas of the brain in SCZ remains to be clarified.

It is possible that *RasGRP1* expression changes in DLPFC of SCZ patients affect downstream clinical phenotypes. For example, such changes may support specific clinical domains of the disorder, such as negative and cognitive symptoms, particularly implicating the DLPFC but not the hippocampus, possibly via the dopamine D1 receptor. Notably, we found that, in patients with SCZ, RasGRP1 protein expression in DLPFC is inversely correlated with age. Again, the specific biological valence of this finding is not fully apparent, but it is possible that the dysfunctional effect of *RasGRP1* expression on DLPFC phenotypes of SCZ is prominent at earlier stages of SCZ pathophysiology and become progressively less important with aging. Further investigation is necessary in order to ascertain this hypothesis.

This is an observational study, performed on a relatively small number of post-mortem brain and blood samples and with relatively small effects detected in differences between SCZ patients and controls post-mortem DLPFC and serum RasGRP1 levels, not providing any replication of both post-mortem and in vivo results.

Therefore, our study provides very preliminary results on the topic and does not indicate any conclusive mechanistic insights on the role of RasGRP1 in pathophysiology of SCZ. We are also aware that the main limitation of our study is that our post-mortem brain and serum samples had incomplete information about medication assumption in patients with SCZ, thus not allowing for any result adjustment for this variable’s confounding effect. Nonetheless, while our results were corrected for a number of other potential confounders, including age and gender, to our knowledge, this is the first work contemporaneously exploring brain and peripheral blood expression of the RasGRP1 gene in SCZ. Our findings strongly encourage further investigation in a larger cohort of patients from different ethnicity in order to corroborate our preliminary evidence of a potential use of RasGRP1 protein level in the blood serum as a biomarker of SCZ.

## Figures and Tables

**Table 1:** Demographic and clinical characteristics of control subjects and schizophrenia patients.

Characteristics	Control	Schizophrenia	Statistic	*p*-Value
Subjects (total number)	20	20	−	−
Gender (M/F)	16/4	12/8	χ^2^ = 1.071 (*df* = 1)	0.301 ^a^
Age (years, median [IQR])	73.50 [66.00–80.25]	52.50 [39.50–61.25]	*t* = 4.819 (*df* = 38)	<0.001 ^b^
PMI (hours, median [IQR])	12.90 [11.80–16.32]	15.25 [12.52–24.58]	*t* = −2.426 (*df* = 38)	0.020 ^c^
pH (median, [IQR])	6.54 [6.49–6.63]	6.50 [6.42–6.56]	*t* = 0.708 (*df* = 26)	0.485 ^c^
RIN (median, [IQR])	6.05 [5.50–7.12]	6.75 [6.05–7.08]	*t* = −0.036 (*df* = 38)	0.971 ^b^

Abbreviations: M/F: number of males/females; PMI: post-mortem interval; RIN: RNA integrity number; IQR: Interquartile Range (i.e., first-third quartiles); *df*: degrees of freedom. ^a^ *p*-value from Chi-Square test (with Yates’s correction); ^b^ *p*-value from two sample *t*-test; ^c^ *p*-value from two sample *t*-test on log transformed values.

**Table 2:** Demographic and clinical characteristics of control subjects and patients with SCZ in the Bari cohort.

Characteristics	Control	Schizophrenia	Statistic	*p*-Value
Subjects (total number)	39	40	−	−
Gender (M/F)	15/24	22/18	χ^2^ = 2.17 (*df* = 1)	0.141
Age (years, median [IQR])	28 [24,25,28–32]	33.50 [27.25–54.5]	*t* = 4.021 (*df* = 78)	<0.001

Abbreviations: M/F: number of males/females; IQR: Interquartile Range (i.e., first-third quartiles); *df*: degrees of freedom. For each variable, statistical tests adopted to detect any significant difference between patients with schizophrenia and control subjects along with respective *p*-values are also reported.

## Data Availability

The data that support the findings of this study are available from the corresponding author upon reasonable request.

## Supplementary Materials

The following supporting information can be downloaded at: https://www.mdpi.com/article/10.3390/biom12020328/s1, Table S1: Demographic characteristics, comorbidities, clinical diagnosis of each control subject and schizophrenia patient.
