# Supplementary material for: Abnormal RasGRP1 Expression in the Post-Mortem Brain and Blood Serum of Schizophrenia Patients
Source: Biomolecules. 2022 Feb 18;12(2):328. doi: 10.3390/biom12020328 (PMC8869509; doi:10.3390/biom12020328)
Supplement: Supplementary file 1 [file biomolecules-12-00328-s001.zip › biomolecules-1544910-supplementary.pdf]

Supplemental Information for

Abnormal RasGRP1 expression in the post-mortem brain and blood serum of schizophrenia patients.

Arianna De Rosa[1,#], Anna Di Maio[1,#], Silvia Torretta[2], Martina Garofalo[1,3], Valentina Giorgelli[2], Rita Masellis[2], Tommaso Nuzzo[1,3], Francesco Errico[1,5,], Alessandro Bertolino[2,5], Srinivasa Subramaniam[6], Antonio Rampino[2,5,*] and Alessandro Usiello[1,3,*]

[1] Laboratory of Translational Neuroscience, CEINGE Biotecnologie Avanzate, 80145, Naples, Italy, derosaar@ceinge.unina.it (A.D.R.); dimaio@ceinge.unina.it (A.D.M.); garofalom@ceinge.unina.it (M.G.); nuzzo@ceinge.unina.it (T.N.); erricof@ceinge.unina.it (F.E.)

[2] Group of Psychiatric Neuroscience, Department of Basic Medical Sciences, Neuroscience and Sense Organs, University of Bari Aldo Moro, 70124, Bari, Italy, silvia.torretta@uniba.it (S.T.), valegio3@gmail.com (V.G.); rita.masellis@uniba.it (R.M.); antonio.rampino@uniba.it (A.R.)

[3] Department of Environmental, Biological and Pharmaceutical Science and Technologies, Università degli Studi della Campania "Luigi Vanvitelli", 81100, Caserta, Italy; usiello@ceinge.unina.it (A.U.)

[4] Department of Agricultural Sciences, University of Naples "Federico II", Naples, Italy; francesco.errico@ unina.it (F.E.)

[5] Azienda Ospedaliero-Universitaria Consorziale Policlinico, 70124, Bari, Italy; alessandro.bertolino@uniba.it (A.B.)

[6] Department of Neuroscience, The Scripps Research Institute, Florida, Jupiter, Florida, 33458, USA.; SSubrama@scripps.edu., (S.S)

[#] Share the Co-First authorship

[*] Correspondence: usiello@ceinge.unina.it; Tel.: +39-0813737899 (A.U.); antonio.rampino@uniba.it; Tel.: +39 080 5478588 (A.R.)

Table S1. Demographic characteristics, comorbidities, clinical diagnosis of each control subject and schizophrenia patient.

Control						Schizophrenia						
ID	*Sex*	*Age (years)*	*PMI (hours)*	*pH*	*Clinical Diagnosis*	*ID*	*Sex*	*Age (years)*	*PMI (hours)*	*pH*	*Clinical Diagnosis*	*Antipsychotic medication*
1	M	47	12.5	6.53	CA (esophagus) with metastases to the liver	*21*	M	46	21.7	6.45	Schizophrenia, Depression, Bipolar, Seizure disorder, Epilepsy, Substance abuse	Quietapine
2	M	66	17.3	6.49	CA (lung), COPD	*22*	M	55	10.7	N.A.	Schizophrenia, Suicide, Overdose, Depression, Anxiety, Hypochondriasis	Risperidone, Fluphenazine
3	F	92	23.3	6.75	CA (uterus, stomach), Congestive hearth failure, Hypertension, Macular degeneration,	*23*	M	53	20.5	N.A.	Schizophrenia, Depression, Bipolar, CA (lung) Paranoia, Psychosis, Hypertension, Anxiety	Olanzapine, Fluphenazine
4	M	84	11.8	6.79	CA (stomach), Renal failure, acute, COPD	*24*	M	70	24.0	6.56	Schizophrenia, Paranoia, Aggressive behavior, Dementia, Impulse disorder, Tuberculosis	N.A.
5	M	70	11.8	6.62	Coronary hearth disease, Leukemia, Type I diabetes, Myocardial infarction, Congestive	*25*	M	35	35.7	6.51	Schizophrenia, Alcohol abuse	Risperidone, Haloperidol
6	M	87	9.3	6.76	Congestive hearth failure, Atherosclerosis, COPD	*26*	F	32	12.3	6.51	Schizophrenia, Alcohol abuse history	N.A.
7	M	58	9.0	6.32	CA (colon)	*27*	M	61	28.0	6.73	Schizophrenia, Aggressive behavior, Suicide, Attempts, Anxiety, Tobacco abuse, Asthma	Thioridazine, Fluphenazine, Quietapine, Paliperidone
8	M	68	10.5	N.A.	CA (lung), Alcohol abuse, Type I diabetes, Transient Ischemic Attack	*28*	F	41	20.8	6.39	Schizophrenia, Suicide, Stabbing, Psychosis, Disassociated Disorders, Electroconvulsive	Quietapine, Haloperidol, Risperidone
9	M	80	14.0	6.49	CA (bladder), Hypertension, Diabetes type II, CVA	*29*	F	50	13.7	N.A.	Schizophrenia, Suicide, Overdose, Aggressive behavior, Depression, Migraine, Hallucination	Risperidone
10	M	76	16.0	6.55	CA (lung). Pulmonary emphysema	*30*	M	18	26.3	6.72	Schizophrenia, Mentally retarded (clinical only), Attention Deficit Disorder, Sleep Apnea	Risperidone
11	M	75	11.5	6.6	CA (prostate) Coronary hearth disease, Hypertension	*31*	M	46	11.6	6.41	Schizophrenia, Suicide, hanging, Alcohol abuse, Substance abuse (not Alcohol), Depression	Quietapine, Risperidone
12	M	66	13.3	N.A.	CA (larynx) Metastasis to bone and liver, Type I diabetes	*32*	F	29	27.3	6.4	Schizophrenia, Depression, Seizure Disorder, Attention Deficit Disorder, Aggressive behavior	Aripiprazole, Risperidone
13	M	64	17.5	6.63	Lymphoma, Coronary Artery Disease	*33*	M	60	10.3	N.A.	Schizophrenia, Depression, Bipolar, Alcohol abuse, Psychotic disorder, Inappropriate sexual	Ziprasidone, Risperidone
14	M	80	12.0	N.A.	CA (kidney) Hypertension, Atrial fibrillation, Macular degeneration, COPD	*34*	F	75	14.9	N.A.	Schizophrenia, CA (pancreas), Dementia, Therapeutic lobotomy, Dysphagia	Risperidone
15	F	83	17.6	6.41	CA (breast, uterus, colon), Macular degeneration, Chronic urinary tract infection	*35*	F	77	14.7	6.38	Schizophrenia, Alcohol abuse, Depression, Bipolar and Seizure disorder	Fluphenazine, Risperidone
16	F	79	14.0	N.A.	Coronary hearth disease, Hypertension	*36*	M	77	26.5	6.56	Schizophrenia, Hypertension, Dementia, COPD	Quietapine
17	M	61	19.5	6.29	Normal	*37*	M	24	12.8	6.47	Schizophrenia, Depression, Substance abuse (not Alcohol), Suicide Attempts, Alcohol abuse	Aripiprazole, Quietapine
18	M	70	12.0	N.A.	Renal failure, acute, Diabetes type I	*38*	F	62	12.2	6.67	Schizophrenia, Stroke/CVA, Depression, Hypertension, Diabetes type I	Quietapine
19	M	72	12.2	6.54	COPD, Pulmonary emphysema	*39*	F	52	15.6	6.49	Schizophrenia, Depression, CA (pancreas), Diabetes Type II, Hypothyroidism, Hypertension	Compazine, Risperidone
20	F	81	14.5	N.A.	COPD, Pneumonia, Osteoporosis, Tuberculosis	*40*	M	55	12.6	N.A.	Schizophrenia, Paranoid Schizophrenia, Infection Bacterial, Psychotic disorder, Hallucination	Fluphenazine, Chlorpromazine

Abbreviations: ID: Identifier (progressive) number for each recruited subject; PMI: post-mortem interval; M: male; F: female; CA: carcinoma; COPD: chronic obstructive pulmonary disease; CVA: cerebrovascular accident; N.A.: Not Available.
